# Validation of reliability, repeatability and consistency of three-dimensional choroidal vascular index

**DOI:** 10.1038/s41598-024-51922-x

**Published:** 2024-01-18

**Authors:** Feiyan Ma, Yifan Bai, Jialiang Duan, Yuchen Liang, Qingli Shang

**Affiliations:** The Second Hospital of Hebei Medical University, 215 Heping Road, Shijiazhuang, Hebei Province China

**Keywords:** Image processing, Eye diseases

## Abstract

This study aimed to investigate the reliability, repeatability and consistency of choroidal vascularity index (CVI) measurements provided by an artificial intelligence-based software in swept-source optical coherence tomography (SS-OCT) in normal subject, and to evaluate the influencing factors for 3D-CVI. Repeatability of 3D-CVI by SS-OCT was evaluated based on different scanning modes including Macular Cubes (3 mm × 3 mm, 6 mm × 6 mm, 9 mm × 9 mm) and Optic Nerve Head 6 mm × 6 mm. Intraclass Correlation Coefficient (ICC) was used to estimate the repeatability and reproducibility of five repeated measurement by SS-OCT. Consistency of CVI between SS-OCT and spectral-domain optical coherence tomography (SD-OCT) was measured and compared in a pilot study of ten eyes and agreement between SS-OCT and SD-OCT was evaluated by Bland–Altman analysis and Deming regression. The influencing factors for 3D-CVI including age, gender, axial length and spherical equivalent on CVI was further investigated in a prospective study of 125 eyes of 125 healthy subjects. ICC between different measurements by SS-OCT was 0.934 (95% CI 0.812–0.956) indicating good repeatability. Intraclass correlation coefficient between CVI measure by SS-OCT and SD-OCT was 0.887 (95% CI 0.796–0.938, *P* value < 0.001). The mean difference between 3D-CVI measured by SS-OCT and SD-OCT 0.133. CVI measured with SS-OCTA showed stronger correlations with axial length and age but not correlated with gender. There is good agreement between CVIs obtained from the built-in software that requires less timing in manual quantification. Studies investigating choroidal vascularity can be standardized by the AI-based CVI analyze software.

## Introduction

The choroid is located between the retina and the sclera and consists of five layers, namely Bruch’s membrane, choriocapillaris, Haller’s layer, Sattler’s layer, and the suprachoroid^1^. The main task of the choroid was to provide oxygen and nutrients to the outer retina. As one of the most highly vascularized tissues in the body, the choroid plays an extremely important role in the physiological processes of the eye. Changes to the choroidal structure have been associated with a variety of choroidal retinal diseases, such as age-related macular degeneration (AMD), polypoidal choroidal vasculopathy (PCV), central serous chorioretinopathy (CSC), Vogt-Koyanagi-Harada disease (VKH), and diabetic retinopathy (DR)^2^.

Quantified choroidal parameters have been applied in clinical evaluation for choroidal disorders, including sub-foveal choroid thickness (SFCT), choroidal volume (CV), choroidal vascularity index (CVI)^3,4^. SFCT have been extensively explored in various clinical settings. However, SFCT is considered an one-dimensional parameter and CV failed to differentiate choroidal vascularity from choroidal matrix. Choroidal vascularity has gained prominant interest in clinical application^5^. The term ‘choroidal vascularity index (CVI)’ was introduced to denote the ratio of luminal area (LA) to the total choroidal area (TCA), with a higher CVI indicating a greater proportion of vascular tissue compared to stromal tissue in the choroid, as proposed by Agrawal et al.^6^.Compared with SFCT, CVI exhibits less variability and remains unaffected by physiological variables such as age, axial length, intraocular pressure, and blood pressure, rendering it a more reliable indicator for evaluating choroidal structure.

The primary approach to quantify CVI involves capturing choroidal images with high resolution using enhanced depth imaging optical coherence tomography (EDI-OCT). Subsequently, image processing software such as ImageJ has to be used to binarize the images and manually or automatically mark the choroidal boundary to obtain the CVI value^7,8^. However, even with EDI-OCT, the light scattering of the retinal pigment epithelium (RPE) to the choroid significantly impacts measurement acuracy, especially in cases of pachychoroid, making it challenging to identify the outer boundary of the choroid. Compared with spectral domain optical coherence tomography (SD-OCT), swept source optical coherence tomography (SS-OCT) with a wavelength of 1050 nm, offers enhanced penetrability and reduced attenuation through the RPE. This capability allows for better visualization of the entire choroid layer and more precise measurement of choroidal structure^9^.

CVI has been found extensive application in exploring diverse choroidal and retinal conditions^10–12^. Yet, most CVI analyses was rely on a two-dimensional choroidal vascular index calculated from a single optical coherence tomography (OCT) B-scan through the fovea or a selected location. However, due to the topographical changes of the choroid, this index cannot fully represent the changes in the choroidal vascular structure^13^. Therefore, leveraging the three-dimensional or volumetric information of the choroid becomes imperative for a more accurate assessment of choroidal vessels, advocating for the three-dimensional choroidal vessel index (3D-CVI)^14^.

Even though previous studies have investigated CVI in both healthy individuals and those with ocular pathologies such as diabetic retinopathy (DR), central serous chorioretinopathy (CSCR), age-related macular degeneration (AMD), posterior uveitis, and retinitis pigmentosa (RP) based on SS-OCT^2,15,16^, the reliability for this parameter remained unassessed in a comprehensive manner in previous research. The purpose of the study is to validated repeatability and reproducibility of 3D-CVI from SS-OCT, consistency with the CVI obtained with SD-OCT that later processed with standard protocol, and related factors affecting 3D-CVI in healthy objects.

## Results

### Demographics

125 eyes of 125 subjects were enrolled with 10 eyes included to assess the reliability, reproducibility, and consistency of CVI measurements using SS-OCT. For the investigation of factors influencing CVI, comprising 60 males and 65 females. The age of the subjects ranged from 6 to 70 years old, with a mean age of (38.27 ± 15.06) years old. The axial length of the subjects' eyes ranged from 21.14 mm to 28.11 mm, with a mean axial length of (23.80 ± 2.43) mm.

### Intra-observer repeatability of 3D-CVI

Macular Cube 6 mm * 6 mm, 9 mm * 9 mm, and ONH 6 mm * 6 mm showed excellent reproducibility between different measurements (ICC > 0.95, *P* < 0.001) in terms of repeatability and reliability. Angio 3 * 3 showed good reliability (ICC > 0.7, *P* < 0.001), shown in Table 1.

**Table 1:** 3D-CVI repeatability quantified by SS-OCT in different scanning modes.

Scanning mode	Number of measurements	$\overline{x }$ ± s	ICC	ICC 95%CI	*P*
Angio 3 * 3	1	0.4 ± 0.09	0.708	0.426–0.919	< 0.001
	2	0.42 ± 0.11			
	3	0.41 ± 0.11			
	4	0.44 ± 0.1			
	5	0.42 ± 0.12			
Angio 6 * 6	1	0.41 ± 0.09	0.980	0.947–0.995	< 0.001
	2	0.42 ± 0.11			
	3	0.42 ± 0.1			
	4	0.42 ± 0.11			
	5	0.42 ± 0.1			
Angio 9 * 9	1	0.38 ± 0.08	0.973	0.927–0.994	< 0.001
	2	0.39 ± 0.08			
	3	0.38 ± 0.09			
	4	0.38 ± 0.08			
	5	0.38 ± 0.08			
ONH Angio 6 * 6	1	0.32 ± 0.03	0.978	0.927–0.994	< 0.001
	2	0.35 ± 0.08			
	3	0.32 ± 0.04			
	4	0.33 ± 0.05			
	5	0.32 ± 0.02			

### Inter-observer reliability of 3D-CVI based on SS-OCTA and SD-OCT

**Figure 1:**
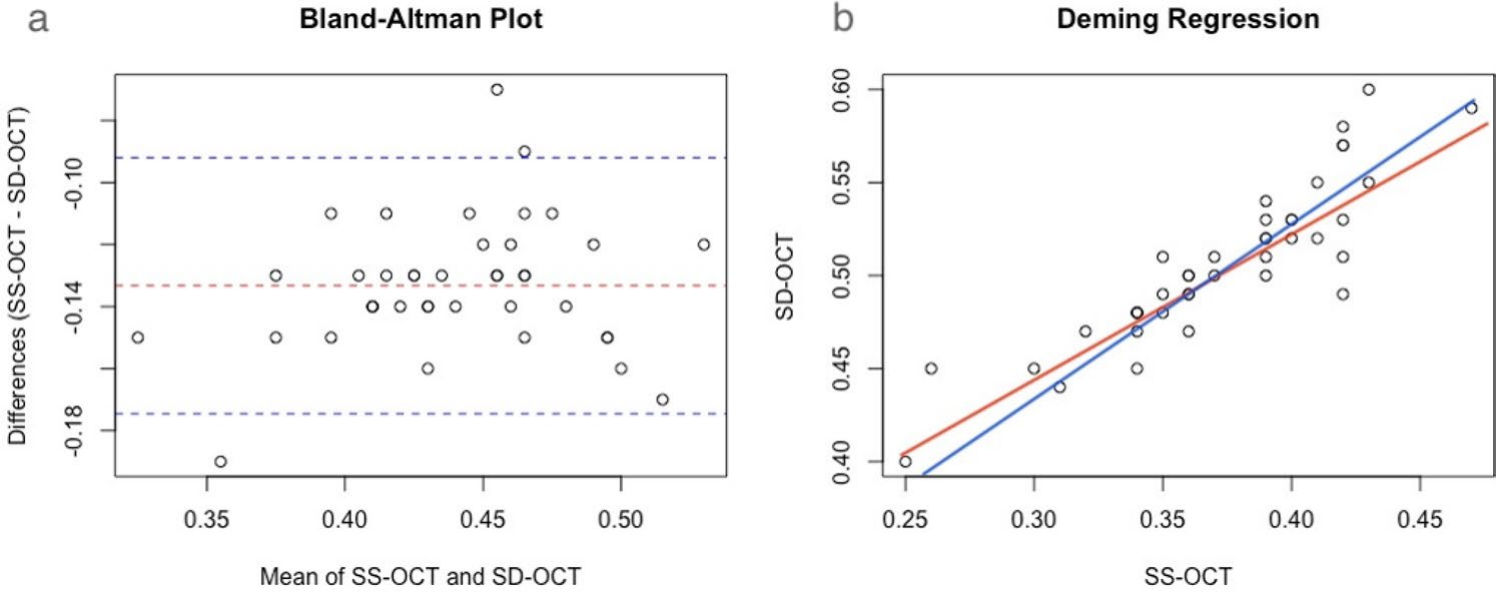
Agreement assessment of 3D-CVI between SS-OCT and SD-OCT. (**a**) Bland–Altman plots showing mean differences of 3D-CVI between SS-OCT and SD-OCT was less than 0.15. (**b**) Deming regression plot of 3D-CVI between SS-OCT and SD-OCT in healthy study population. The plots illustrate the fitted linear models (red line) and the identity lines (SS-OCT measurement = SD-OCT measurement, slope = 1) (blue line). Intercepts and slopes of the fitted linear model are shown as mean (95% confidence interval) and indicate excellent agreement of the 3D-CVI scan measurements between SS-OCT and SD-OCT.

The inter-observer reliability of CVI measurements from SS-OCTA showed good consistency between that obtain by SD-OCT with an ICC of 0.887 (0.796–0.938, *P* < 0.001). Average CVI based on SS-OCTA was 0.37 ± 0.05 while that based on SD-OCT was 0.51 ± 0.04. Bland–Altman showed good consistency between the two parameters (Fig. 1). The Bland–Altman plot of SS-OCT and SD-OCT demonstrated a normal distribution; the mean difference of CVI was 0.133 (0.507 for SS-OCT and 0.373 for SD-OCT) and 95% of the measurements fell within 2SD of the mean. The Bland–Altman plot of SS-OCTA1 and SD-OCT measurements also showed that the 95% limits of agreement for differences fell within 10% of the mean of the measurements (0.092–0.175 with a mean difference of 0.133), indicating good agreement between the two pairs of variables (t = 39.81, *P* = 0.00).

### Influencing factors for 3D-CVI

#### Gender

CVIs in all partitions (S, T, I, N) were not significantly different between male and female (*P* > 0.05) in all scanning modes (Macular Cube 3 mm * 3 mm,6 mm * 6 mm,9 mm * 9 mm and ONH 6 mm * 6 mm). There were no significant differences in 3D-CVI measurements by Macular Cube 3 mm * 3 mm and 6 mm * 6 mm in different gender groups. In the Macular Cube 9 mm * 9 mm, there was a statistically significant difference in 3D-CVI measurements in the temporal region of the macula between different gender groups (*P* = 0.01), while there was no statistical difference in 3D-CVI measurements in other regions of Macular Cube 9 mm * 9 mm. In the ONH 6 mm * 6 mm, there was a statistically significant difference in 3D-CVI measurements in the nasal region, while there was no statistical difference in 3D-CVI measurements in other regions. Detailed data were shown in Table 2.

**Table 2:** 3D-CVI in each partition of different scanning modes over the macula and optic nerve.

Scanning mode	Partition	Male	Female	Z./t	*P*
Angio 3 * 3	0–3 mm, $\overline{x }$ ± s	0.39 ± 0.10	0.38 ± 0.10	0.26	0.795
	0–1 mm, $\overline{x }$ ± s	0.41 ± 0.05	0.40 ± 0.08	− 0.585	0.559
	S (1–3 mm), $\overline{x }$ ± s	0.40 ± 0.07	0.40 ± 0.10	− 0.239	0.811
	T (1–3 mm), $\overline{x }$ ± s	0.34 ± 0.11	0.35 ± 0.11	− 0.33	0.742
	I (1–3 mm), $\overline{x }$ ± s	0.40 ± 0.10	0.41 ± 0.11	− 0.207	0.836
	N (1–3 mm), $\overline{x }$ ± s	0.39 ± 0.11	0.36 ± 0.12	1.12	0.265
Angio 6 * 6	0–6 mm, $\overline{x }$ ± s	0.35 ± 0.04	0.35 ± 0.03	− 1.265	0.206
	0–1 mm, $\overline{x }$ ± s	0.37 ± 0.06	0.38 ± 0.04	− 0.377	0.706
	S (1–6 mm), $\overline{x }$ ± s	0.35 ± 0.05	0.35 ± 0.03	− 0.179	0.858
	T (1–6 mm), $\overline{x }$ ± s	0.32 ± 0.06	0.34 ± 0.06	− 1.389	0.167
	I (1–6 mm), $\overline{x }$ ± s	0.34 ± 0.06	0.36 ± 0.06	− 1.608	0.110
	N (1–6 mm), $\overline{x }$ ± s	0.36 ± 0.07	0.37 ± 0.07	− 0.749	0.455
Angio 9 * 9	0–9 mm, $\overline{x }$ ± s	0.31 ± 0.04	0.32 ± 0.04	− 1.456	0.148
	0–1 mm, $\overline{x }$ ± s	0.31 ± 0.04	0.32 ± 0.04	− 1.435	0.151
	S (1–9 mm), $\overline{x }$ ± s	0.31 ± 0.05	0.32 ± 0.05	− 1.019	0.310
	T (1–9 mm), $\overline{x }$ ± s	0.29 ± 0.05	0.31 ± 0.04	− 2.609	0.010*
	I (1–9 mm), $\overline{x }$ ± s	0.32 ± 0.04	0.33 ± 0.05	− 1.176	0.242
	N (1–9 mm), $\overline{x }$ ± s	0.31 ± 0.05	0.31 ± 0.05	− 0.682	0.496
ONH Angio 6 * 6	0–6 mm, $\overline{x }$ ± s	0.32 ± 0.03	0.32 ± 0.02	− 0.859	0.390
	0–1 mm, $\overline{x }$ ± s	0.01 ± 0.01	0	− 0.107	0.915
	S (1–6 mm), $\overline{x }$ ± s	0.33 ± 0.04	0.33 ± 0.03	− 1.019	0.308
	T (1–6 mm), $\overline{x }$ ± s	0.34 ± 0.04	0.35 ± 0.04	− 0.021	0.983
	I (1–6 mm), $\overline{x }$ ± s	0.33 ± 0.05	0.33 ± 0.03	− 0.513	0.608
	N (1–6 mm), $\overline{x }$ ± s	0.34 ± 0.04	0.31 ± 0.05	− 2.311	0.021*

#### Age

**Figure 2:**
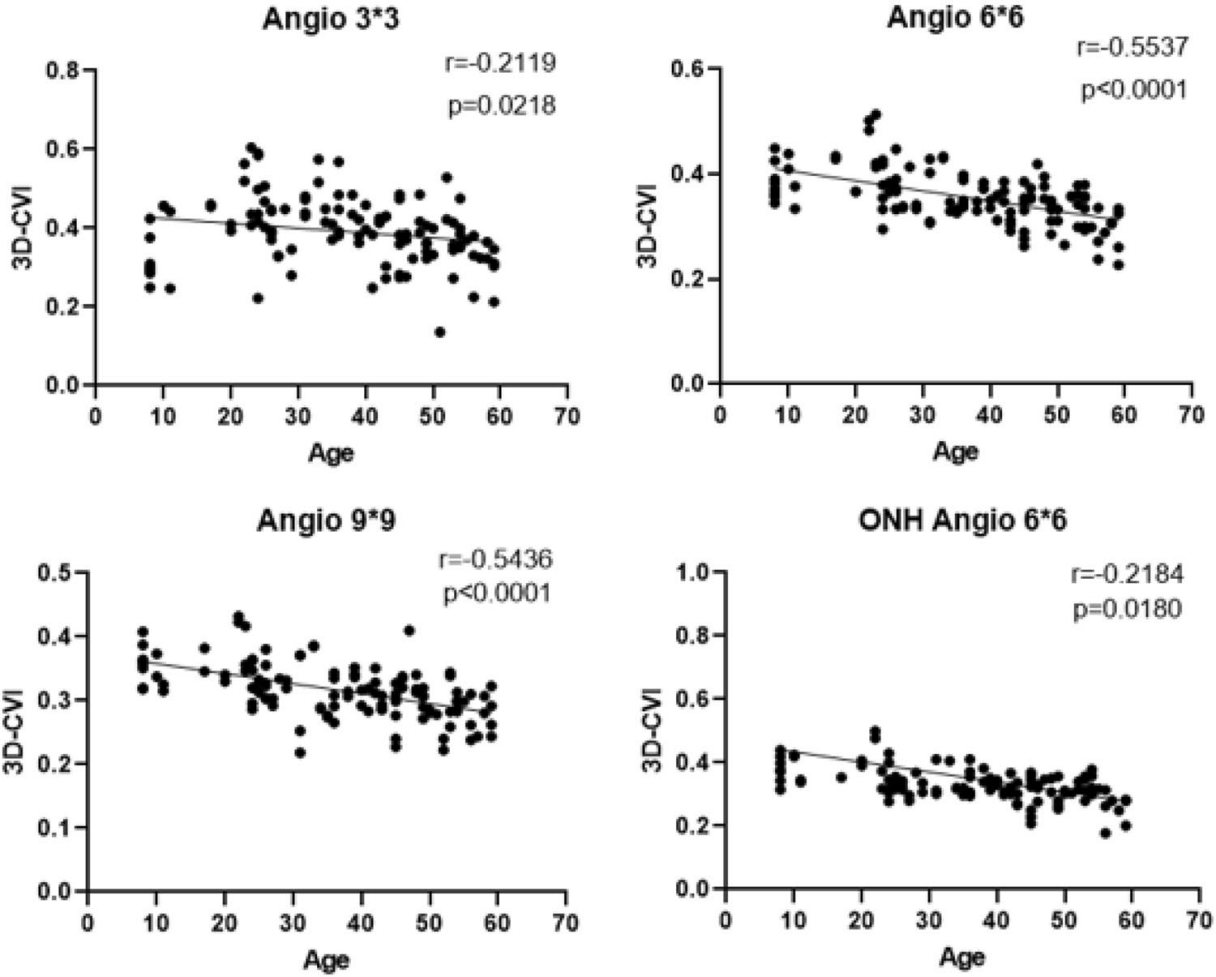
The correlation between age and 3D-CVI by scanning with macular fovea as the center and with CVI on optic nerve.

In Macular Cube 3 mm * 3 mm, there was a weak negative correlation between 3D-CVI and age (r = 0.2119, *P* = 0.0218). In the Macular Cube 6 mm * 6 mm and 9 mm * 9 mm, there was a strong negative correlation between 3D-CVI and age(*P* < 0.0001). In ONH 6 mm * 6 mm, there were significant statistical differences in the 3D-CVI among different age groups, and the 3D-CVI showed a weak negative correlation with age. The correlation between age and CVI were showed in Fig. 2. Multiple linear regression analysis indicates a negative correlation between age and 3D-CVI in Macular Cube 3 mm * 3 mm (β = − 0.114, *P* = 0.001), Macular Cube 6 mm * 6 mm (β = − 0.547, *P* = 0.00), Macular Cube 9 mm * 9 mm (β = − 0.503, *P* = 0.000), and ONH 6 mm * 6 mm (β = − 0.581, *P* = 0.000).

#### Axial length

**Figure 3:**
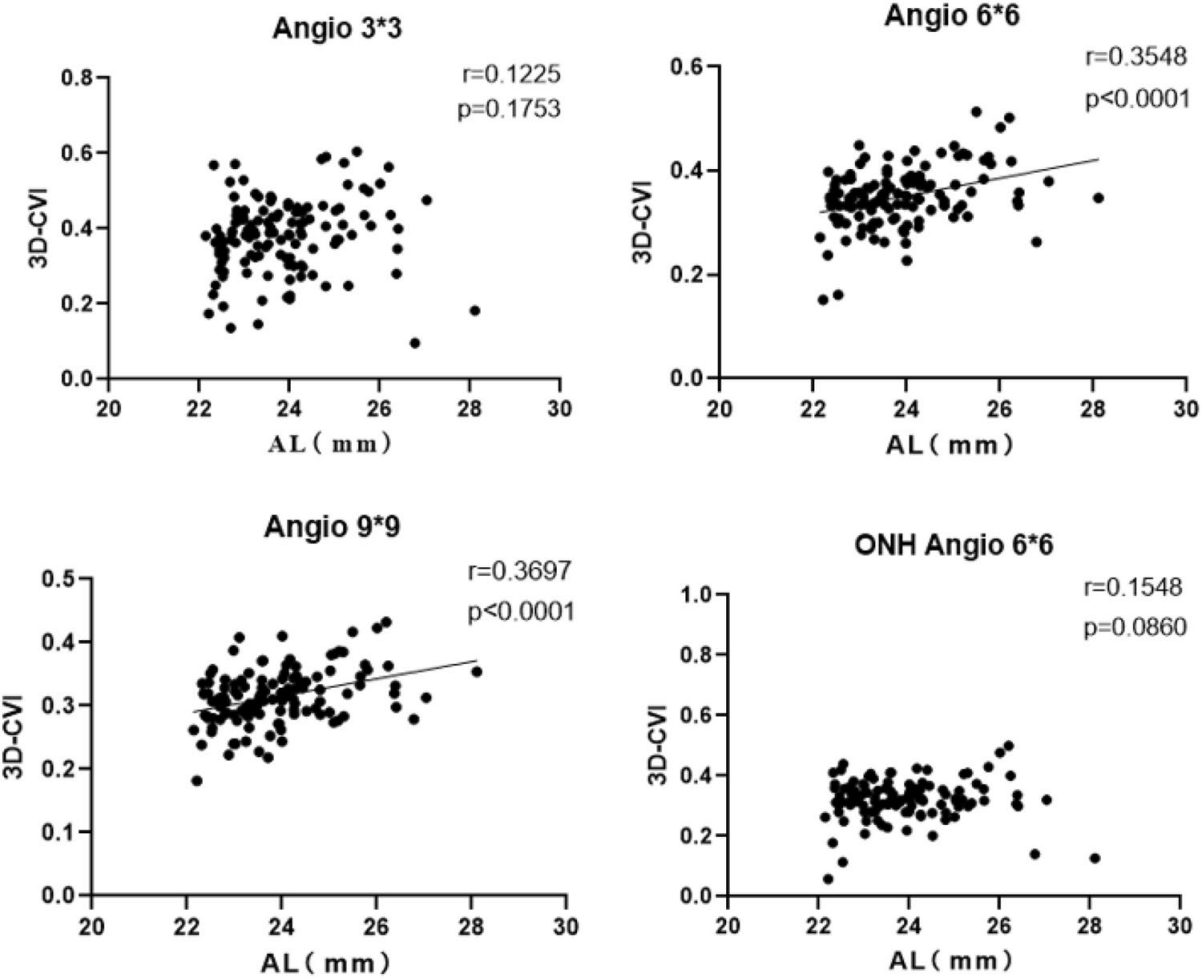
The correlation between axial length and 3D-CVI by scanning with macular fovea as the center and with CVI on optic nerve.

There were statistically significant differences in 3D-CVI among the different axial length groups across the three macular cube modes. There was no correlation between 3D-CVI and axial length in the Macular Cube 3 mm * 3 mm. In the Macular Cube 6 mm * 6 mm and 9 mm * 9 mm, there was a positive correlation between 3D-CVI and axial length. In the ONH Angio 6 * 6 scanning mode, there was no correlation between 3D-CVI and axial length, and there was no significant difference in data acroos the various axial length groups. The correlated between age and CVI were showed in Fig. 3. Multiple linear regression analysis indicates a positive correlation between age and 3D-CVI in Macular Cube 6 mm * 6 mm (β = 0.182, *P* = 0.013), Macular Cube 9 mm * 9 mm (β = 0.218, *P* = 0.004).

## Discussion

This study employed SS-OCT to gather extensive sets of normal choroidal images. Leveraging the device's built-in deep learning algorithm, it automatically delineated choroidal boundaries and quantified the CVI within the scanning area. This approach proves more convenient than the traditional method, which involves using local adaptive thresholding to binarize the segmented choroidal images, reconstructing the choroid in 3D, and subsequently calculating CVI through binarization. Despite the current application of 3D-CVI in disease quantification analysis, there remains an absence of comparative analysis regarding the consistency of this parameter across different algorithms. Our study focused on analyzing and comparing the consistency of 3D-CVI obtained from different algorithms, alongside exploring the correlation between normal 3D-CVI and gender, age, and axial.

To the best of our knowledge, this study stands as the first to compare the consistency between the 3D-CVI automatically quantified by the SS-OCT built-in deep learning algorithm and the results obtained through traditional quantification methods of EDI-OCT. The findings revealed strong consistency (ICC: 0.887, 95% CI 0.796–0.938, *P* < 0.001), suggesting the reliability of data derived from artificial intelligence. This reliability proves proves beneficial for monitoring pathological changes in clinical practice and significantly alleviates the burden of quantified image analysis. Furthermore, the device demonstrated good reproducibility, indicating the stability of its quantitative outcomes. This stability facilitates diagnosis and treatment of diseases in clinical settings.

Whether gender should be considered an influencing factor for CVI has been disputable. The study identified statistical differences between gender in the temporal region of the fovea in the Macular Cube 9 mm * 9 mm scanning as well as in nasal area in ONH Angio 6 mm * 6 mm. In a study by Goud A et al.^5^, encompassing 30 healthy participants (19 females), ETDRS partition centered at the macula was utilized, with inner ring diameters of 1 mm, middle ring (1–3 mm), and outer ring (3–6 mm). CVI was computed for each B-scan using the EDI mode of SD-OCT, and subsequently 3D-CVI of each region was quantified. The findings from this study indicated no significant difference between genders. The variances in different results might be attributable to several factors.

Firstly, our study included a larger sample size and utilized SS-OCT for scanning, offering improved penetration compared to SD-OCT. This allowed for clearer imaging of the outer boundary of choroid, enhancing the viability for image analysis. Secondly, in Goud A et al.’s study, 3D-CVI was calculated based on 31 scans using SD-OCT, with a scan spacing of 3.87 μm, potentially resulting in a relative loss of choroidal volume between scanning lines. Thirdly, while both studies employed the ETDRS partition, they differed in partitioning techniques. Our study segmented the images obtained from each scanning mode into fovea and surrounding S, T, I, N areas.

The results of this study demonstrated a significant negative correlation between overall 3D-CVI and age across different scanning modes (Angio 3 * 3 r = − 0.2119, *P* = 0.0218; Angio 6 * 6 r = − 0.5537, *P* < 0.0001; Angio 9 * 9 r = − 0.5436, *P* < 0.0001; ONH Angio 6 * 6 r = − 0.2184, *P* = 0.018). Notably, in the Macular Cube 3 mm * 3 mm, the 3D-CVI quantification of the Temporal, Inferior, and Nasal regions exhibited weak correlation with age. Conversely, in other scanning modes, the quantification of 3D-CVI for each subregion (S, T, I, N) showed strong negative correlation with age. Goud et al.’s study also reported a negative correlation between 3D-CVI and age (r = − 0.384, *P* = 0.03)^2^. However, in Cheong et al.’s study (n = 30), which quantified the 3D-CVI within a 30°circle centered on the fovea, no correlation was found between 3D-CVI and age (*P* = 0.081)^12^. Similarly, Sun G et al.'s study quantified the 3D-CVI of 63 healthy subjects' choroids within a 3 * 3 mm range centered on the fovea and found no correlation between 3D-CVI and age (r = 0.043, *P* = 0.741)^17^.

Our study results differed from prior reports. For instance, Guduru et al. observed a significant correlation disc perimeter CVI and age based on SS-OCT (Topcon) in 29 healthy subjects (58 eyes)^18^. Yet, focusing solely on 2D-CVI acquired through linear scans or single measurement centered on the fovea might not be adequately unveil changes in the overall state of the choroid. Our study addresses this limitation by illustrating alterations across the entire three-dimensional structure of the macular or optic disc area. In contrast to Nivison-Smith et al.’s findings indicating a decrease in 2D-CVI with age, particularly pronounced in the inferior hemisphere retina^19^, our study did not align with these changes. This disparity could be attributed to our quantification methodology, which leveraged a three-dimensional volume area, providing a more precise depiction of topographic changes across the entire posterior pole. The negative correlation discovered between 3D-CVI and age suggests a decline in choroidal blood flow as individuals age. This decline may compromise the ability of the choroid to deliver oxygen and other metabolites to the retinal pigment epithelium (RPE) and retina. This finding partially elucidates the onset of age-related macular degeneration^5^.

The results from our study revealed a positive correlation between the overall quantitative 3D-CVI of Macular Cube 6 mm * 6 mm and 9 mm * 9 mm regions and the axial length (Angio 6 * 6 r = 0.3548, *P* < 0.0001; Angio 9 * 9 r = 0.3697, *P* < 0.001). However, no correlation was observed between the quantitative 3D-CVI of the Macular Cube 3 mm * 3 mm and the ONH 6 mm * 6 mm region and the axial length.

Xu et al. conducted a study involving 113 myopic patients, evaluating their 3D-CVI within the Angio 6 mm * 6 mm centered on the macula using SS-OCT (VG100S). They discovered a negative correlation with axial length (*P* < 0.05)^20^. Additionally, they observed that the degree of myopia exhibited a negative correlation with choroidal blood flow. In Luo H et al.'s study, 135 subjects were categorized into four groups based on axial length, and the 3D-CVI and 3D-CVV of the Angio 6 * 6 region centered on the macula were quantified for each group. Their study showed a negative correlation between 3D-CVI, 3D-CVV, and axial length (3D-CVI, r = − 0.3667, *P* < 0.0001; 3D-CVV, r = − 0.5284, *P* < 0.0001). These results mark a significant difference from previous studies, hinting at the possibility that as axial length increases, the loss in choroidal matrix might surpass the decline in choroidal vascular volume. Alshareef et al. investigated structural changes of the inferior choroid vessels among individuals with myopia and observed a substantial reduction in matrix composition with the elongation of axial length compared to individuals without myopia^21^. Similarly, though Yazdani et al.’s study quantified in 2D-CVI, they arrived at the same conclusion, noting that the 2D-CVI in the myopic group exceeded that of the normal group.

### Limitation of the study

This study solely on quantifying 3D-CVI in healthy eyes devoid of underlying diseases. Moreover, further investigations is warranted to understand the impact of refractive error on 3D-CVI. Larger-scale studies are essential to validate the findings derived from this research.

## Methods

### Study population and study design

The study was designed as prospective observational study and adhere to the principles of the Declaration of Helsinki. Approval for the study was obtained from the Human Research and Ethics Committee of The Second Hospital of Hebei Medical University, and written consent was acquired from all participants before enrollment. Recruitment was conducted between October 2022 and January 2023. Inclusion criteria involved individuals aged between 6 and 70 years, with best corrected visual acuity (BCVA) of or better than 25/25 (Snellen visual acuity) and refractive error ranging from − 6D to + 6D with, with a cutoff signal strength index value ≥ 6 as the imaging control criteria. Exclusion criteria included (1) individuals with refractive media opacity or insufficient ability to fixate, (2) eyes with abnormalities including glaucoma, uveitis, diabetic retinopathy, age-related macular degeneration, el al; (3) ocular history involving trauma or intraocular surgery, (4) presence of strabismus or amblyopia, and (5) eyes with imaging quality less than 6 assessed by the software.

All eligible participants underwent standardized ophthalmologic examination including measurements of spherical equivalent (SE), BCVA, intraocular pressure (IOP), and axial length (AL). The measurements were carried out between 4 and 5 pm to minimize the potential impact of diurnal ocular variations on the findings.

### SS-OCTA imaging acquisition and Analysis of 3D-CVI

In this study, the VG200D SS-OCT system by Svision Micro-Image Technology Co., Ltd. was used with a 1050 nm central wavelength, 200,000 A-scan/sec scanning speed, and 9 mm tissue scanning depth. All scans, including Macular Cube 3 mm × 3 mm, 6 mm × 6 mm, 9 mm × 9 mm, and ONH 6 mm × 6 mm modes, at 512 × 512 resolution, were conducted by the same experienced physician between 3 and 5 pm to mitigate potential diurnal rhythm effects.

**Figure 4:**
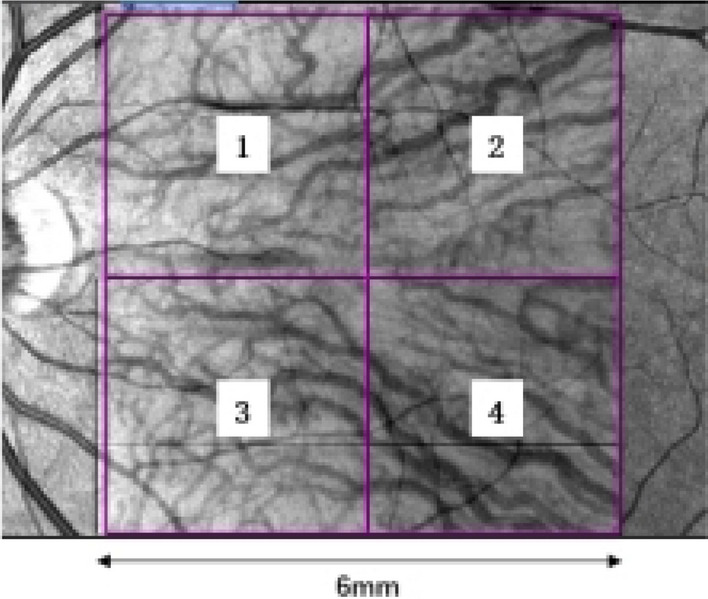
The image on the left depicts infrared imaging of the fundus obtained via SS-OCT. The 6 * 6 mm range, centered on the fovea, is highlighted by a purple box and is further divided into four parts using a 3 * 3 mm grid, denoted as 1–4.

For measurement repeatability study, five measurements without tracking reference were performed for the same eye; For consistence study of 3D-CVI measurements between SD-OCT and SS-OCT, four partitions within the macula cubic 6 mm * 6 mm area was overlaid onto the fundus image (Fig. 4). For analyzing influencing factors for 3D-CVI, the Early Treatment Diabetic Retinopathy Study (ETDRS) grid with three concentric circles with diameters of 1 mm, 3 mm, and 6 mm were superimposed on the fundus image, centered on both the fovea and optic disc. Additionally, the parafoveal and peripapillary regions were subdivided into superior (S), inferior (I), nasal (N), and temporal (T) quadrants.

Three-D CVI is defined as the ratio of CVV (Choroidal Vascular Volume) divided by the total volume of the measurement region. All influencing factors for determining 3D-CVI, namely the area size of the large blood vessel lumens within the choroid and the total choroidal volume are directly obtained from SS-OCT raw images. Built-in equipment with deep-learning algorithms was employed for the automatic segmentation of the inner and outer boundaries of the choroid. These boundaries were subsequently verified and corrected manually by a qualified physician. The 3D-CVI is calculated in the following steps in SS-OCT as previously described^22^. Briefly, OCTA fundus images were captured using a scanning protocol of 512 horizontal B-scans, encompassing a region of approximately 6 mm × 6 mm at the fovea's center. These B-scans, each consisting of 512 A-scans, underwent four repetitions and were then averaged. The choriocapillaris layer was defined as a segment extending from the base of the retinal pigment epithelium-Bruch’s membrane complex to 20 μm behind it, within the choroid itself. To identify regions lacking flow within the choriocapillaris, a global thresholding method utilizing standard deviation values from a normal database (known as the SD method) was applied to each choriocapillaris image. Layer segmentation algorithms in SS-OCT was based on Deep Learning Approaches. Specifically, the images underwent semi-automated segmentation and binarization using Niblack’s autolocal threshold, employing custom algorithms developed in MATLAB R2017a (MathWorks, Natick, MA, USA).

### SD-OCT imaging acquisition and analysis of 3D-CVI obtained on SD-OCT

**Figure 5:**
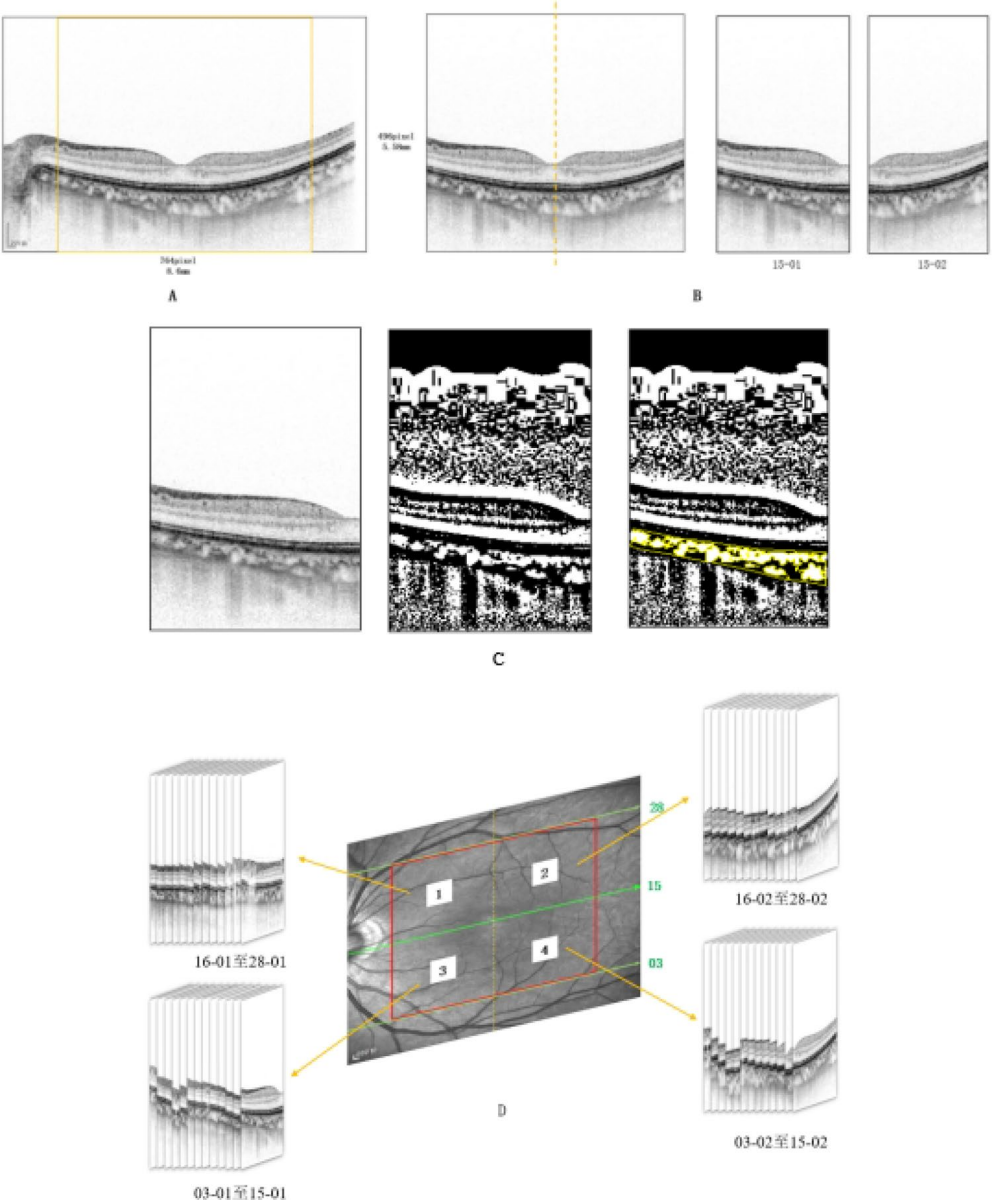
The b-scan image was obtained through the fovea with a size of 8.6 mm * 5.58 mm (764 pixel * l496 pixel). Since the image required for the study is within a 6 mm range, the required range is selected accordingly (indicated by the yellow box in the figure). To further analyze the image, we take the fovea as the boundary and divide it into two parts, which are labeled as 15-01 and 15-02. We then apply ImageJ software for binary processing on 15-01 and use the formula to calculate the CVI of the region. Finally, the figure shows examples of all partitions.

In SD-OCT, 31 scanning lines were applied for 3D-CVI analysis as in previous report^6^. Briefly, the choroid was imaged using the EDI mode of SD-OCT (Spectralis, Heidelberg Engineering, Heidelberg, Germany). The macular region was scanned using a 31 horizontal line scan (6 mm × 6 mm) centered on the fovea, with 100 frames averaged in each B-scan. Each scan was spaced 200 μm apart from each other. The obtained images underwent binarization process with segmentation following the method outlined by Sonoda et al.^8^. Binarization of the image was conducted utilizing publicly available software, ImageJ (version 1.47; http://imagej.nih.gov/ij/) (Fig. 5).

### Statistical analysis

The normality of the data was assessed with the Shapiro–Wilk test. Independent sample t-test was used for the data with uniform variance and the t' test was used for the data with uneven variance. Categorical variables are described using median (upper and lower quartiles) (M(P25, P75)). Data reproducibility and consistency were calculated using intra-group correlation coefficient (ICC). For ICC, values less than 0.5 indicate poor reliability, values between 0.5 and 0.75 indicate moderate reliability, values between 0.75 and 0.9 indicate good reliability, and values greater than 0.90 indicate excellent reliability. Mann–Whitney U test was used for comparison of differences between groups. Univariate linear regression analyses were performed to examine the associations among ocular and systemic factors with CVI. Age and factors that were significant in the univariate analyses (*P* < 0.05) were included in the multivariable regression model. Statistical significance level was determined at a critical value of *P* < 0.05.

### Ethics approval and consent to participate

The study protocol was approved by the Second Hospital of Hebei Medical University (Approval No. 2021-R100) and conducted in accordance with the principles outlined in the Declaration of Helsinki. Written informed consent was obtained from all participants prior to their inclusion in the study.

## Data Availability

The datasets generated and/or analyzed during the current study are available from the corresponding author upon reasonable request. The raw data, including imaging files and measurement data, will be made available to researchers for the purpose of replication and further analysis.
